# Vitamin D analogs combined with 5-fluorouracil in human HT-29 colon cancer treatment

**DOI:** 10.3892/or.2014.3247

**Published:** 2014-06-11

**Authors:** MAGDALENA MILCZAREK, BEATA FILIP-PSURSKA, WIESŁAW ŚWIĘTNICKI, ANDRZEJ KUTNER, JOANNA WIETRZYK

**Affiliations:** 1 Department of Experimental Oncology, Ludwik Hirszfeld Institute of Immunology and Experimental Therapy, Polish Academy of Sciences, 53-114 Wroclaw, Poland; 2 Wroclaw Research Centre EIT+, 54-066 Wroclaw, Poland; 3 Pharmaceutical Research Institute, 01-793 Warsaw, Poland

**Keywords:** vitamin D analogs, colon cancer, combined treatment, 5-fluorouracil

## Abstract

In the present study, we evaluated the antitumor effect of two synthetic analogs of vitamin D, namely PRI-2191 [(24R)-1,24-dihydroxyvitamin D_3_] and PRI-2205 (5,6-trans calcipotriol), in combined human colon HT-29 cancer treatment with 5-fluorouracil (5-FU). Mice bearing HT-29 tumors transplanted subcutaneously or orthotopically were injected with vitamin D analogs and 5-FU in various schedules. A statistically significant inhibition of subcutaneous or orthotopic tumor growth was observed as a result of combined therapy. In HT-29 tumors and in cells from *in vitro* culture, we observed increased vitamin D receptor (VDR) expression after treatment with either PRI-2205 or 5-FU alone, or in combination. Moreover, PRI-2205 decreased the percentage of cells from intestinal tumors in G_2_/M and S stages and increased sub-G_1_. Increased VDR expression was also observed after combined treatment of mice with 5-FU and PRI-2191. Moreover, our docking studies showed that PRI-2205 has stronger affinity for VDR, DBP and CAR/RXR ligand binding domains than PRI-2191. PRI-2191 analog, used with 5-FU, increased the percentage of subcutaneous tumor cells in G_0_/G_1_ and decreased the percentage in G_2_/M, S and sub-G_1_ populations as compared to 5-FU alone. In *in vitro* studies, we observed increased expression of p21 and p-ERK1/2 diminution via use of both analogs as compared to use of 5-FU alone. Simultaneously, PRI-2191 antagonizes some pro-apoptotic activities of 5-FU *in vitro*. However, in spite of these disadvantageous effects in terms of apoptosis, the therapeutic effect expressed as tumor growth retardation by PRI-2191 is significant. Our results suggest that the mechanism of potentiation of 5-FU antitumor action by both analogs is realized via increased p21 expression and decreased p-ERK1/2 level which may lead to diminution of thymidylate synthase expression. Higher binding affinity for VDR, DBP, but also for CAR\RXR ligand binding domain of PRI-2205 may, in part, explain its very low toxicity with sustained anticancer activity.

## Introduction

According to clinical and epidemiological studies, vitamin D and calcium may reduce the risk of colorectal cancer via various mechanisms (1–7). Moreover, the impact of vitamin D or its analogs on colon cancer cell proliferation, apoptosis, differentiation and cell cycle regulation is being investigated. The regulation of gene expression by vitamin D and its analogs progresses via binding to specific vitamin D receptors (VDR) upon ligand activation and dimerization with retinoid X receptors (RXR). Target genes possess specific nucleotide sequences: vitamin D response elements (VDREs) which bind VDR-RXR heterodimers to activate or suppress their expression (8–10). Post-transcriptional regulatory mechanisms of gene expression have also been proposed (11). The expression of VDR is low in normal colonic epithelial cells, but increases with malignant transformation and then decays with progressive tumor growth. This phenomenon is correlated with a decreasing level of VDR in the nucleus as compared with the cytoplasm. At the same time, high VDR expression correlates with an advantageous prognosis in colorectal patients, suggesting an important role for VDR in the pathogenesis of colon cancer (1,6,7,12).

Vitamin D deficiency in mice has been shown to result in the aggressive growth of mouse MC-26 colon cancer (13). Other experimental data have shown that dietary vitamin D significantly reduced the incidence of colonic tumors in rats or mice treated with the carcinogen (14,15). Moreover, deletion of the VDR gene in mice alters the balance between proliferation and apoptosis, increases oxidative DNA damage and enhances susceptibility to carcinogenesis (16).

**Figure 1:**
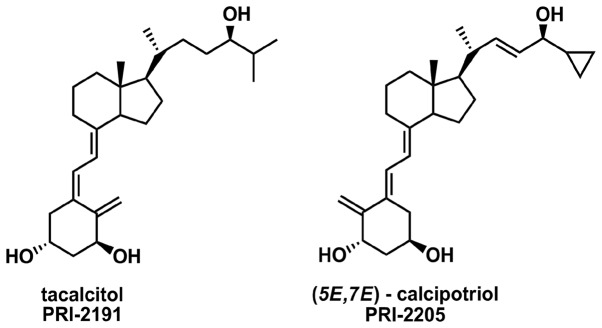
Chemical structures of vitamin D analogs tacalcitol (PRI-2191) and PRI-2205, (5E,7E)-isomer of calcipotriol.

Studies on combined treatment with 1,25(OH)_2_D_3_ or its analogs and different chemotherapeutic agents have been reported *in vitro* (17–22) and *in vivo* (23,24). Previously, we examined the biological activity against various cancer and normal cell lines of a series of side-chain modified and diastereomeric and geometric analogs of vitamin D (21,25,26). We also evaluated the influence of vitamin D analogs on the activity of a range of anticancer drugs *in vitro* and *in vivo* against the human and murine cancer cells (19–21,25–30). On the basis of these results, we selected two analogs for further studies: PRI-2191 (tacalcitol, 1,24-dihydroxyvitamin D_3_) and PRI-2205 (5,6-trans calcipotriol) (chemical structures are shown in Fig. 1). The selected analogs reveal higher antitumor and lower calcemic activity as well as lower toxicity than 1,25(OH)_2_D_3_ (26,28).

In the present study, we analyzed the effect of the vitamin D analogs PRI-2191 and PRI-2205 on the antitumor activity *in vivo* of 5-fluorouracil (5-FU) in mice bearing human (HT-29) colon cancer. The mechanism of action of the anti-metabolite agent 5-FU includes its ability to induce the level and activity of the tumor suppressor gene *p53* and to stabilize p53 protein (31,32). Moreover, it has been shown that the target gene of p53 - *p21*^w^*^af1/cip1^* is a primary 1,25(OH)_2_D_3_-responding gene with VDR binding promoter regions, in which p53 also co-localizes (10). In this connection, the 1,25(OH)_2_D_3_ can induce the expression of p21 independently on the activity of p53 protein, what consequently leads to the cell cycle arrest and inhibition of cell proliferation. This fact is important especially when the colon cancer cells exhibit mutated p53 protein, such as for example the HT-29 cell line used in our studies. Previous studies have shown that the human parathyroid calcium-sensing receptor (CaSR) is expressed in human colon epithelium and regulates epithelial proliferation and differentiation. Moreover, 1,25(OH)_2_D_3_ is involved in regulation of CaSR expression (33–35). Notably, 1,25(OH)_2_D_3_, as well as calcipotriol, promoted the sensitivity of human colon carcinoma cells to anticancer drugs, including 5-FU, which may be mediated through the CaSR (36,37). These data suggest that combined therapy with the use of vitamin D analogs and 5-FU may be promising.

## Materials and methods

### Compounds

1,25(OH)_2_D_3_ (calcitriol), PRI-2191, calcipotriol (PRI-2201) and PRI-2205 were obtained as certified synthetic materials from the Pharmaceutical Research Institute, Warsaw, Poland. Samples of the compounds were stored in amber ampoules, under argon at −20°C. Prior to usage, in the case of *in vitro* studies, compounds were dissolved in 99.8% ethanol to the concentration of 10^−4^ M and subsequently diluted in culture medium in order to reach the concentration of 100 nM. For animal experiments, compounds were dissolved in 99.8% ethanol, then diluted in 80% propylene glycol in order to reach the required concentrations and administered subcutaneously (s.c.) to mice in a volume of 5 μl/1 g of body weight.

5-FU (ICN Polfa, Rzeszów, Poland) solution at a concentration of 50 mg/ml was diluted in culture medium prior to usage in *in vitro* studies in order to reach the required concentrations and for *in vivo* experiments in saline in order to reach the required concentrations and then administered either intravenously (i.v.) or intraperitoneally (i.p.) to mice at a volume of 10 μl/1 g of body weight.

Capecitabine (CPC) (Pharmaceutical Research Institute, Warsaw, Poland) was dissolved in 40% ethanol, then diluted in water for injection in order to reach the required concentration and administered orally (p.o.) to mice at a volume of 10 μl/1 g of body weight.

### Cells

The human colon cancer cell line HT-29 was obtained from the German Cancer Research Center (Deutsches Krebsforschungszentrum, DKFZ, Heidelberg, Germany) the origin of the cell line, Leibniz Institute DSMZ-German Collection of Microorganisms and Cell Cultures, Braunschweig, Germany. The cell line was cultured *in vitro* at the Cell Culture Collection of the Institute of Immunology and Experimental Therapy, Wroclaw, Poland.

### Mice

The mice, female 6–8-week old NOD/SCID, Nu/J and NCr-nu/nu mice, weighing 20–25 g, were supplied by the University Children’s Hospital in Krakow (Poland) and the Medical University of Bialystok (Bialystok, Poland), Charles River Labs., National Cancer Institute, Frederic, USA respectively. Mice were maintained in specific pathogen-free (SPF) conditions. All animal experiments were performed according to EU Directive 2010/63/EU for animal experiments and were approved by the 1st Local Committee for Experiments with the Use of Laboratory Animals, Wroclaw, Poland.

### Design of the in vivo experiments

Human colon cancer HT-29 cells were harvested with the use of 0.05% trypsin/0.02% EDTA, washed twice with serum-free minimum essential medium (α-MEM) and resuspended in Hank’s medium. A single-cell suspension (3.5×10^6^/200 μl per mouse) with cell viability >90% was inoculated subcutaneously (s.c.). For orthotopic transplantation (i.i.), the anesthetized mouse was placed on a wooden board in the right lateral position and the incision was made through the left upper abdominal pararectal line and peritoneum. The cecal wall was carefully exposed, placed and fixed between layers of sterile gauze. A 1–2 mm piece of specimen, derived from a primary tumor grown s.c. in another mouse, was fixed to the serosal part of the cecal wall with 5-0 surgical sutures. After implantation, the peritoneum and abdominal wall were sutured with 4-0 surgical sutures (Dexon-‘S’; Polfa, Poznań, Poland). Tumor cell transplantations were performed under general anesthesia with a mixture of ketamine hydrochloride (100 mg/kg; Ketamina 10%; Biowet, Puławy, Poland) and xylazine hydrochloride (20 mg/kg; XylaRiem; Riemser Arzneimittel AG, Germany).

### Details of the treatment schedules used

#### Determination of the dose and scheme of 1,25(OH)_2_D_3_ analog treatment

The treatment of NOD/SCID mice bearing subcutaneous HT-29 tumors was started either on day 12 (Fig. 4A left graph) or on day 5 (Fig. 4A right graph).

In the first experiment, 5-FU was administered intravenously (i.v.) at a dose of 75 mg/kg/day once a week for 4 weeks (on days 12, 19, 26, 33; total dose, 300 mg/kg). PRI-2191 or PRI-2205 were injected s.c. at doses of 0.2 μg/kg/day or 5.0 μg/kg/day, respectively, 5 times a week on days 12, 13, 14, 15, 16, 19, 20, 21, 22, 23, 26, 27, 28, 29, 30, 33, 34, 35 (total dose of PRI-2191, 3.6 μg/kg; PRI-2205, 90 μg/kg). This experiment was ended on day 36 and, after cell inoculation, the tumors were harvested for further analyses.

In the second experiment, 5-FU was administered intravenously (i.v.) at a dose of 75 mg/kg/day once a week, for 5 weeks (on days 5, 12, 19, 26, 33; total dose, 375 mg/kg). PRI-2191 or PRI-2205 were injected s.c. at doses of 1.0 μg/kg/day or 10.0 μg/kg/day, respectively, 3 times a week on days 7, 10, 12, 14, 17, 19, 21, 24, 26, 28, 31, 33, 35, 38, 40, 42, 45, 47 (total dose of PRI-2191, 18 μg/kg; PRI-2205, 180 μg/kg). This experiment was ended on day 49 and, after cell inoculation, the tumors were harvested for further analyses.

#### Treatment after orthotopic transplantation

The treatment of NCr-nu/nu mice bearing orthotopic HT-29 tumors was started either on day 17 (Fig. 5A left graph) or on day 11 (Fig. 5A right graph).

In the first experiment, 5-FU was administered i.v. at a dose of 75 mg/kg/day once a week, for 4 weeks (on days 17, 24, 31, 38; total dose, 300 mg/kg). PRI-2191 or PRI-2205 were injected s.c. at doses of 1 μg/kg/day or 10 μg/kg/day, respectively, 3 times a week on days 17, 19, 21, 24, 26, 28, 31, 33, 35, 38, 40, 42, 45, 47 (total dose of PRI-2191, 14 μg/kg; PRI-2205, 140 μg/kg). This experiment was ended on day 53 after cell inoculation. The blood was also collected and the calcium serum level was analyzed.

In the second experiment, mice were i.p. injected with 75 mg/kg/day 5-FU (on days 11, 18, 25, 32; total dose, 300 mg/kg) and/or vitamin D analog PRI-2205 at a dose of 10 μg/kg/day administered s.c. (3 times a week, on days 11, 13, 15, 18, 20, 22, 25, 27, 29, 32, 34, 36; total dose, 120 μg/kg). The experiment was ended on day 39 and, after cell inoculation, the tumors were harvested for further analyses. The blood was also collected and morphological analyses were performed.

#### PRI-2191 or PRI-2205 used in combined colon cancer treatment with oral 5-FU prodrug CPC

Nu/J mice were s.c. inoculated with human colon cancer HT-29 (data not shown). The treatment was started on day 5 after tumor cell inoculation. CPC was administered orally with the use of gastric tubes at a dose of 450 mg/kg/day 5 times a week (on days 5, 6, 7, 10, 11, 12, 13, 14, 17, 18, 19; total dose, 4.95 g/kg). Analogs PRI-2191 or PRI-2205 were administered s.c., 3 times a week, at doses of 1 mg/kg/day or 10 mg/kg/day, respectively (on days 5, 7, 10, 12, 14, 17, 19, 21, 24, 26, 28, 31, 33, 35, 38, 40, 42; total dose PRI-2191, 17 μg/kg; PRI-2205, 170 μg/kg). The experiment was terminated on day 46.

### Evaluation of the therapeutic effect

Tumor diameters were measured three times a week by caliper. Tumor volume was calculated using the formula: (a^2^ × b)/2, where a, a shorter tumor diameter in mm and b, a longer tumor diameter in mm. Inhibition of tumor growth was calculated from the following formula: tumor growth inhibition (TGI) (%) (TGI) = 100 − [(W_T_/W_C_) × 100], where W_T_ is the median tumor weight of treated mice and W_C_ that of untreated control animals. The mice were also weighed three times a week.

### Evaluation of combination effects

The minimal expected inhibition used to estimate the effect of the combination of two compounds was evaluated using the formula: TGI hypothetical values (HTGI) (%)= 100 − [(100 − E for cytostatic) × (100 − E for 1,25(OH)_2_D_3_ analog)/100] (38). Where E was TGI.

As a result of the comparison of TGI to % HTGI, the type of interactions between two compounds in combined treatment was designated and these could be: (i) synergy, when the experimental value of TGI is greater than HTGI; (ii) additive effect, when the two values are comparable; (iii) subadditive effect, if the experimental TGI value is smaller than the hypothetical, but larger than TGI for cytostatic given alone; (iv) antagonism, if the experimental TGI is smaller than the experimental TGI for cytostatic.

### Blood leukocytes and calcium evaluation

The level of blood leukocytes and serum calcium was measured in each individual blood sample using the following devices: Sysmex K4500SL, serial number F2872, Japan and Olympus AU400, Olympus America, Melville, New York, USA, respectively.

### Design of in vitro experiments for cell cycle distribution, cell death and p53 expression analysis

Cultured HT-29 cells were seeded at a density of 1×10^5^ cells/ml of culture medium on 6-well plates (Corning, NY, USA) at a volume of 2 ml. On the following day, the cells were exposed to compounds for 48 h; vitamin D compounds 100 nM and 5-FU 100 or 200 μg/ml. Ethanol, used as a solvent for all compounds and diluted corresponding to its highest concentration, produced no toxicity.

### Cell cycle analysis

#### Preparation of cells from in vitro culture

After 48 h of incubation, the cells were collected with the use of 0.05% trypsin/0.02% EDTA. The cell suspension was washed once in phosphate-buffered saline (PBS) supplemented with 2% of fetal bovine serum (2% PBS). Then, the cells were suspended in 2% PBS and counted in a hemacytometer.

#### Tissue preparation

HT-29 colon cancer cells from primary tumors were obtained by mincing fresh tumor tissue with a scalpel, passing them through a plasma filter and suspending them in PBS. After centrifugation, the cells were disaggregated with the use of 0.05% trypsin/0.02% EDTA. The cell suspension was washed once with PBS supplemented with 2% of fetal bovine serum (2% PBS). Then, the cells were suspended in 2% PBS and counted in a hemacytometer.

Cells (1×10^6^) were fixed for 24 h in 70% ethanol at −20°C. Then, the cells were washed twice in PBS and incubated with RNAse (8 μg/ml, Fermentas, Germany) at 37°C for 1 h. The cells were stained for 30 min with propidium iodide (PI) (0.5 mg/ml; Sigma-Aldrich Chemie GmbH, Steinheim, Germany) at 4°C and the cellular DNA content was determined using a BD FACSCalibur instrument (Becton-Dickinson, San Jose, CA, USA.) and either ModFit LT 3.0 or WinMDI software.

### Death cell analysis with PI staining (sub-G1 stage)

After 48 h of incubation, the cells were prepared in the same manner as cells for the cell cycle distribution assay described above. Data analysis was performed by flow cytometry using a BD LSRFortessa instrument (Becton-Dickinson). Next, data were analyzed in a BD FACSDiva 6.2 program. The experiment was repeated 4 times.

### Apoptosis determination by Annexin V staining

After 48 h of incubation, the cells were collected using non-enzymatic cell dissociation solution (Sigma-Aldrich Chemie GmbH), washed in PBS supplemented with 1% of fetal bovine serum (1% FBS) and counted in a hemacytometer. The cells (2×10^5^) were washed twice with PBS. Annexin V-FITC (Alexis Biochemicals, San Diego, CA, USA) was diluted to a concentration of 1 mg/ml in binding buffer [Hepes buffer: 10 mM HEPES/NaOH, pH 7.4, 150 mM NaCl, 5 mM KCl, 1 mM MgCl_2_, 1.8 mM CaCl_2_, (IIET, Wroclaw, Poland)] and the cells were suspended in 200 μl of this solution (freshly prepared each time). Then, after 15 min of incubation in the dark at room temperature, PI solution (0.1 mg/ml) was added prior to analysis to give a final concentration of 0.01 mg/ml. Data acquisition was performed by flow cytometry on a BD FACSCalibur instrument (Becton-Dickinson) using the CellQuest program. The data were displayed as a two-color dot plot with FITC-Annexin V (FL1-H, Y axis) vs. PI (FL3-H, X axis). Double-negative cells were live cells, PI+/Annexin V+ were late apoptotic or necrotic cells and PI−/Annexin V+ early apoptotic cells. Data were analyzed in the WinMDI 2.9 program. The experiment was repeated 3 times.

### Mitochondrial membrane potential (Ψmt) determination

Mitochondrial injury was assessed by JC-1 (Sigma-Aldrich) staining. This dye, existing in the cytosol as a monomer, remained unprocessed due to a breakdown of Ψmt and fluoresces green. JC-1 can assume a dimeric configuration in the mitochondria and fluoresces red in a reaction driven by the mitochondrial transmembrane potential (39).

After 48 h of incubation, the cells were collected with the use of 0.05% trypsin/0.02% EDTA. The cell suspension was washed once with 2% PBS. Then, the cells were suspended in 2% PBS and counted in a hemacytometer. The HT-29 cells (5×10^5^) were washed in 2% PBS. Pelleted cells were resuspended in 100 μl of warm cultured medium with the addition of 10 μl JC-1 (the final concentration of JC-1 was 3 μg/ml) and were then incubated for 15 min at 37°C. Next, the cells were washed with 1 ml of 2% PBS and were then resuspended in 300 μl of 2% PBS. Data acquisition was performed by flow cytometry on a BD FACSCalibur instrument (Becton-Dickinson) using the CellQuest program. Next, data were analyzed in WinMDI 2.8.

As a positive control of cells with low potential, we used cells which were incubated for 24 h with valinomycin (Sigma-Aldrich) at a concentration of 1 μM.

### Active caspase 3 analysis

After 48 h of incubation, the cells were collected using non-enzymatic cell dissociation solution (Sigma-Aldrich Chemie GmbH), washed in PBS supplemented with 1% of fetal bovine serum (1% FBS) and counted in a hemacytometer. The cells (2×10^5^ per sample) were washed once with 1% PBS and then suspended in 0.1 ml solution to fixing and permeabilization (BD Pharmingen, CA, USA) and incubated for 30 min at 4°C. After incubation, the cells were washed using buffer with added saponin (Perm/Wash Buffer; BD Pharmingen, CA, USA). Next, the cells were stained with anti-active caspase 3 conjugated with phycoerythrin (PE) (BD Pharmingen, CA, USA) for 50 min in the dark at room temperature. After incubation, the cells were washed using buffer with added saponin and then suspended in 0.3 ml in the same buffer. Data analysis was performed by flow cytometry using an LSRFortessa and FACSCalibur instruments (Becton-Dickinson, San Jose, CA, USA). Next, data were analyzed with the BD FACS Diva 6.2 program. The experiment was repeated 4 times.

### p53 expression analysis

After 48 h of incubation, the cells were collected using non-enzymatic cell dissociation solution (Sigma-Aldrich Chemie GmbH), washed in PBS supplemented with 1% FBS and counted in a hemacytometer. The cells (2×10^5^/sample) were washed once with 1% FBS and then suspended in 0.1 ml solution to fixing and permeabilization (BD Pharmingen, CA, USA) and incubated for 30 min at 4°C. After incubation, the cells were washed using buffer with added saponin (Perm/Wash Buffer; BD Pharmingen, CA, USA). Next, the cells were stained either with anti-p53-PE or with an IgG_1,κ_-PE isotype control (BD Pharmingen) for 50 min in the dark at room temperature. After incubation, the cells were washed using buffer with added saponin and then suspended in 0.3 ml in the same buffer. Data analysis was performed by flow cytometry using a BD LSRFortessa instrument (Becton-Dickinson). Next, data were analyzed with BD FACS Diva 6.2. The experiment was repeated 4 times.

### Western blot analysis

#### Tissue preparation

Specimens of tumor tissue from euthanized animals were collected in liquid nitrogen and stored at −80°C. To determine protein expression via western blot analysis, frozen tumors were mechanically homogenized (Rotilabo, Carl Roth, Karlsruhe, Germany) in RIPA buffer (Sigma-Aldrich Chemie GmbH) supplemented with a complete mixture of phosphatase and protease inhibitors (Sigma-Aldrich Chemie GmbH) and then kept on ice for 45 min. Lysates were cleared via microcentrifugation at 17968 rcf × g for 20 min.

#### Preparation of cells from in vitro culture

Cultured HT-29 cells were seeded to a volume of 6 ml and at a density of 2×10^5^ cells/ml of culture medium on a glass Petri dish. Next, after 24 h of incubation the cells were exposed to the test vitamin D compounds at the concentrations of 100 nM and/or 200 μg/ml 5-FU for 48 h.

Protein concentrations were determined using a protein assay (DC Protein Assay; Bio-Rad Laboratories, Hercules, CA, USA). Equal amounts of protein (25 or 50 μg for detecting VDR; 100 μg for p21, p27; ERK and p-ERK and 25, 50 or 100 μg for β-actin) were separated in a 10% (VDR, ERK1/2, p-ERK1/2, β-actin) or 15% (p21, p27) sodium dodecyl sulfate (SDS) polyacrylamide gel and transferred to either a polyvinylidene difluoride (PVDF) membrane (0.45 μm; GE Healthcare, Amersham, Little Chalfont, UK) or a nitrocellulose membrane (0.22 μm; NitroBind, GE Water and Process Technologies, Osmonics, Hopkins, MN, USA). Protein loading and transfer efficiency were monitored via 0.1% Ponceau S-Red staining. Membranes were blocked overnight (4°C) in 1% blocking reagent (membrane blocking agent; GE Healthcare, Amersham) in PBS. On the following day, the membrane was washed three times (×10 min) with 0.05% PBS/Tween-20 (PBST) and then incubated for 1 h at room temperature with a primary antibody: rabbit anti-VDR, anti-p21, anti-27, anti-ERK1/2 or anti-p-ERK1/2 polyclonal antibody (all from Santa Cruz Biotechnology Inc., Santa Cruz, CA, USA) or rabbit anti-β-actin (Sigma-Aldrich, Poznan, Poland). After incubation, the blot was washed three times with 0.1% PBST and incubated for 1 h with the secondary anti-rabbit immunoglobulins (GE Healthcare, Amersham). The membrane was finally washed three times with 0.1% PBST and incubated for 30 min with a fluorescent substrate for alkaline phosphatase-based detection (ECF; GE Healthcare, Amersham). Fluorescence was detected using a scanner (Typhoon scanner; GE Healthcare, UK). Densitometric analysis of the western blots was carried out using ImageJ 1.46r (National Institutes of Health, Bethesda, MA, USA).

### Molecular modeling

Models of proteins were based on available structural data; vitamin D receptor in complex with vitamin D (PDB code: 1DB1), vitamin D-binding protein (DBP) in complex with 25-hydroxyvitamin D3 and oleic acid (PDB code: 1J7E) and the constitutive androstane receptor/retinoid X receptor (CAR/RXR) complex (PDB code: 1XV9). Structural data were processed with the Schroedinger LLC software suite Protein Preparation Wizard (40) and used for docking ligands prepared with ligand preparation in the GlideXP module (41–43) of the same software. Solvent molecules were removed from the complex unless within the 3.5 Å radius and participating in 3 or more hydrogen bonds. Estimates of binding energies were performed with Molecular Mechanics/Generalized Born Surface Area (MM/GBSA) method (VSGB 2.0 energy model) employing implicit solvation (44). The MMGSA model compares favorably with the more computationally intensive Poisson-Boltzmann method (45–47).

### Statistical analysis

Statistical analysis was performed using STATISTICA version 7.1 (StatSoft, Inc., USA). The assumptions of ANOVA were checked using PP-plots, Shapiro-Wilk’s test and Levene’s test. In the case of violations of the ANOVA assumptions, a nonparametric stratified permutation Kruskal-Wallis overall test either with subsequent multiple comparisons or ANOVA followed by Tukey’s HSD for unequal N or Fisher’s test were used. P-values *<*0.05 were considered to indicate a statistically significant difference.

## Results

### Cell cycle and cell death of HT-29 colon cancer in vitro after incubation with PRI-2191 and PRI-2205 applied alone or in combination with 5-FU

The *in vitro* tests were performed after 48 h incubation of colon cancer cells with vitamin D analogs. 1,25(OH)_2_D_3_ and calcipotriol (PRI-2201) were used as reference compounds. In such experimental conditions, vitamin D compounds did not inhibit proliferation of HT-29 colon cancer cells; however, the tendency to improve the antiproliferative activity of 5-FU was observed (Table I). Cell cycle analysis in cultured HT-29 cells showed an increase in G_0_/G_1_ and a simultaneous decrease in S cell cycle stage by reference compounds or PRI-2191. PRI-2205 did not affect cell cycle distribution in the concentrations used (100 nM). 5-FU used alone at the dose of 200 μg/ml reduced the number of cells in G_0_/G_1_ and increased the number in the G_2_/M phase. Reference vitamin D compounds and PRI-2191 further decreased the cells in G_0_/G_1_ and increased those G_2_/M phase as compared to 5-FU used alone. Moreover, a significant decrease in cells in the S stage was observed. PRI-2205 acts differently from other vitamin D compounds; used in parallel with 5-FU, it increased the number of cells in G_0_/G_1_, S and decreased the number in G_2_/M as compared to 5-FU. Moreover, we observed a tendency for the number of cell deaths to increase when PRI-2205 was used together with 5-FU (Table I).

**Figure 2:**
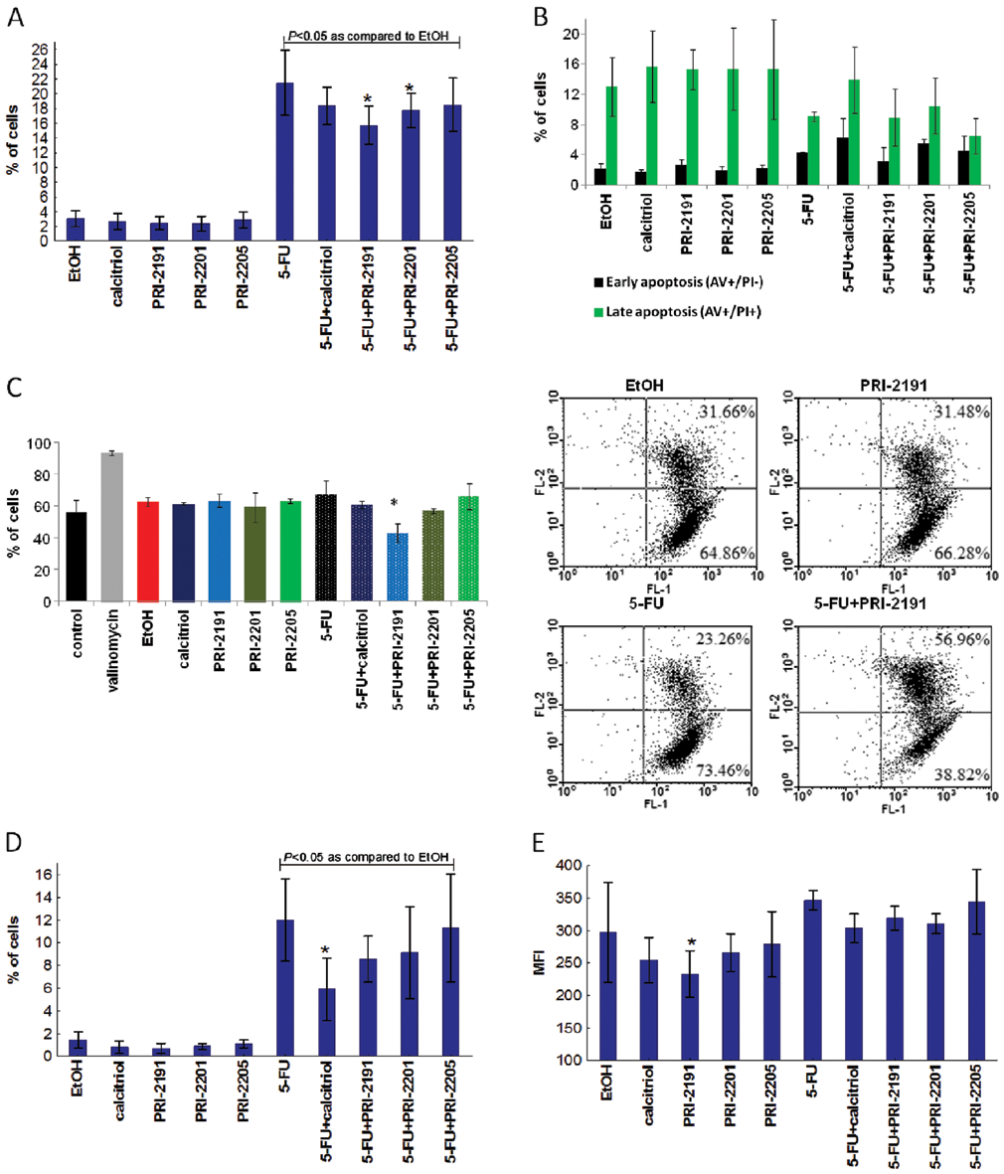
Effect of combined treatment on cell death of colon cancer cells *in vitro*. (A) Propidium iodide (PI)-stained cells, ^*^P<0.05 as compared to 5-FU-treated cells. (B) Annexin V- and PI-stained cells. (C) Mitochondrial membrane potential (Ψmt) of HT-29 cells, ^*^P<0.05 as compared to EtOH-treated cells; Valinomycin-treated cells, P<0.05 as compared to control. Dot-plots from representative experiment for selected groups are presented. (D) Expression of active caspase-3, ^*^P<0.05 as compared to 5-FU-treated cells. (E) Mean fluorescence channel of cells stained with antibodies against p53, ^*^P<0.05 as compared to control. The cells were treated for 48 h with vitamin D compounds at a concentration of 100 nM and with 5-FU; (A,D,E) 100 or (B,C) 200 μg/ml. Calcitriol, 1,25(OH)_2_D_3_; PRI-2201, calcipotriol. Statistical analysis, Fisher’s test. N=4–5 in each group; mean (± SD) is presented. 5-FU, 5-fluorouracil.

In further studies using the same experimental conditions, we analyzed some parameters of the cell death of HT-29 cells (Fig. 2). As shown in Fig. 2A, incubation with 5-FU caused significant HT-29 cell death when counted as positive PI staining. However, simultaneous treatment with vitamin D compounds led to a decrease in the percentage of cell deaths. This phenomenon was very profound when PRI-2191 was used with 5-FU. In apoptotic cells, the externalization of phosphatidylserine is observed from the cytoplasmic to the extracellular membrane site (39). Using Annexin V staining, we studied whether the tested compounds are able to induce apoptotic death and using PI staining, necrotic HT-29 cells were determined. The results are summarized in Fig. 2B. 5-FU, either used alone or in combination with all vitamin D compounds (with the exception of PRI-2191), showed the tendency to increase the number of early apoptotic cells (AV+), as well as to decrease the percentage of late apoptotic cells (AV+/PI+) (Fig. 2B). During apoptosis, the electrochemical gradient across the mitochondrial membrane breaks down (39). In our studies, the influence of the tested compounds on the Ψmt of HT-29 cells was analyzed. However, we did not observe any significant changes in Ψmt independent of the compounds used. Only analog PRI-2191 used with 5-FU caused a decrease in the number of cells with low Ψmt (Fig. 2C).

5-FU alone induced active caspase-3 expression. Analogs used alone had no effect on caspase-3 activity; however, in combination with 5-FU, such analogs reduced the induction of active caspase-3. 1,25(OH)_2_D_3_ diminished active caspase-3 expression to 50% as compared to 5-FU alone. Also, PRI-2191 and PRI-2201 diminished the expression of this enzyme. In contrast, PRI-2205 did not influence the induction of caspase-3 by 5-FU (Fig. 2D).

All vitamin D compounds showed the tendency to decrease p53 expression. Moreover, 1,25(OH)_2_D_3_, PRI-2191 and PRI-2201 decreased the level of p53 as compared to 5-FU alone (Fig. 2E).

**Figure 3:**
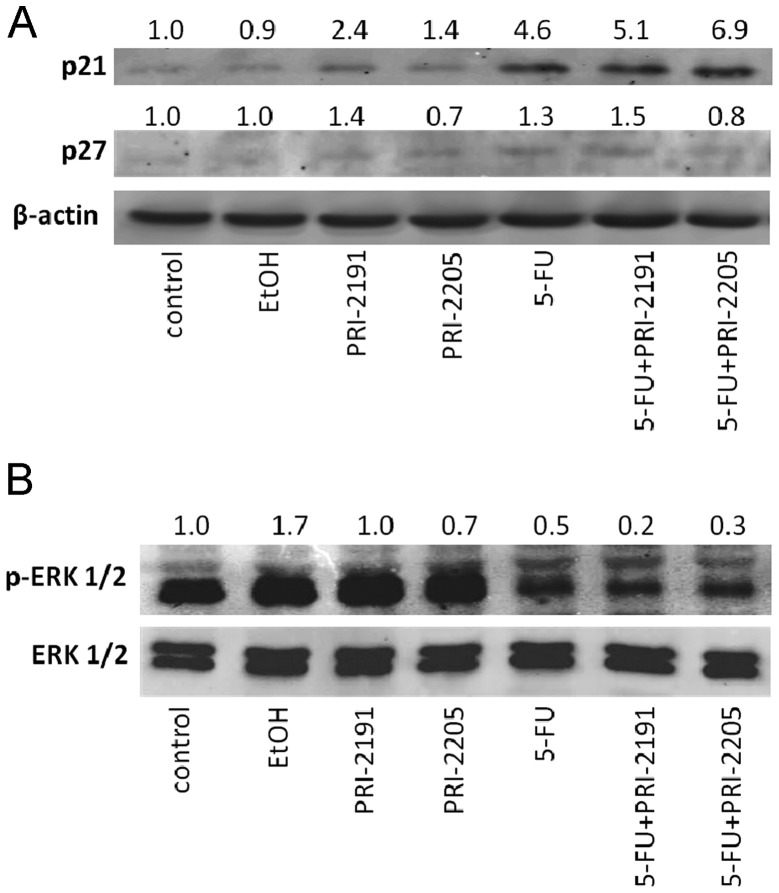
Expression of CDKIs p21 and p27, and ERKs in HT-29 cells. Cultured HT-29 cells were exposed to the vitamin D compounds at a concentration of 100 nM and/or 200 μg/ml of 5-FU for 48 h. Equal amounts of protein (100 μg) were used. Densitometric analysis of the western blots was carried out using ImageJ 1.46r. Calcitriol, 1,25(OH)_2_D_3_; PRI-2201, calcipotriol. CDKIs, cyclin-dependent kinase inhibitors.

A western blot analysis was also performed on the expression of cyclin-dependent kinase inhibitors (CDKI) p21 and p27 (Fig. 3A). The expression of p21 in HT-29 cells was 2.4- and 1.4-fold increased by PRI-2191 and PRI-2205, respectively. 5-FU used alone increased its expression 4.6-fold and a further increase of p21 expression was observed after cell incubation with 5-FU combined with vitamin D analogs. In the case of p27, only a slight induction of this protein expression was observed in cells incubated with either PRI-2191 or 5-FU alone or combined (Fig. 3A).

The level of phosphorylated ERK1/2 (p-ERK1/2) was diminished in cells treated either with 5-FU alone or combined with both PRI-2191 and PRI-2205. The diminution was more profound in cells incubated with 5-FU combined with both analogs (Fig. 3B).

### Effect of PRI-2191 or PRI-2205 administered alone or in combination with 5-FU on subcutaneous human colon cancer HT-29

#### Primary tumor growth

Independent of the treatment schedule, mice treated with PRI-2191 or PRI-2205 developed similar tumors to those in control mice. They also did not affect the body weight of the animals treated (max. 8% decrease). 5-FU decreased tumor volume, but not in a significant manner; TGI was in the range of 15–50%. 5-FU used alone decreased the body weight by ~5% (data not shown).

**Figure 4:**
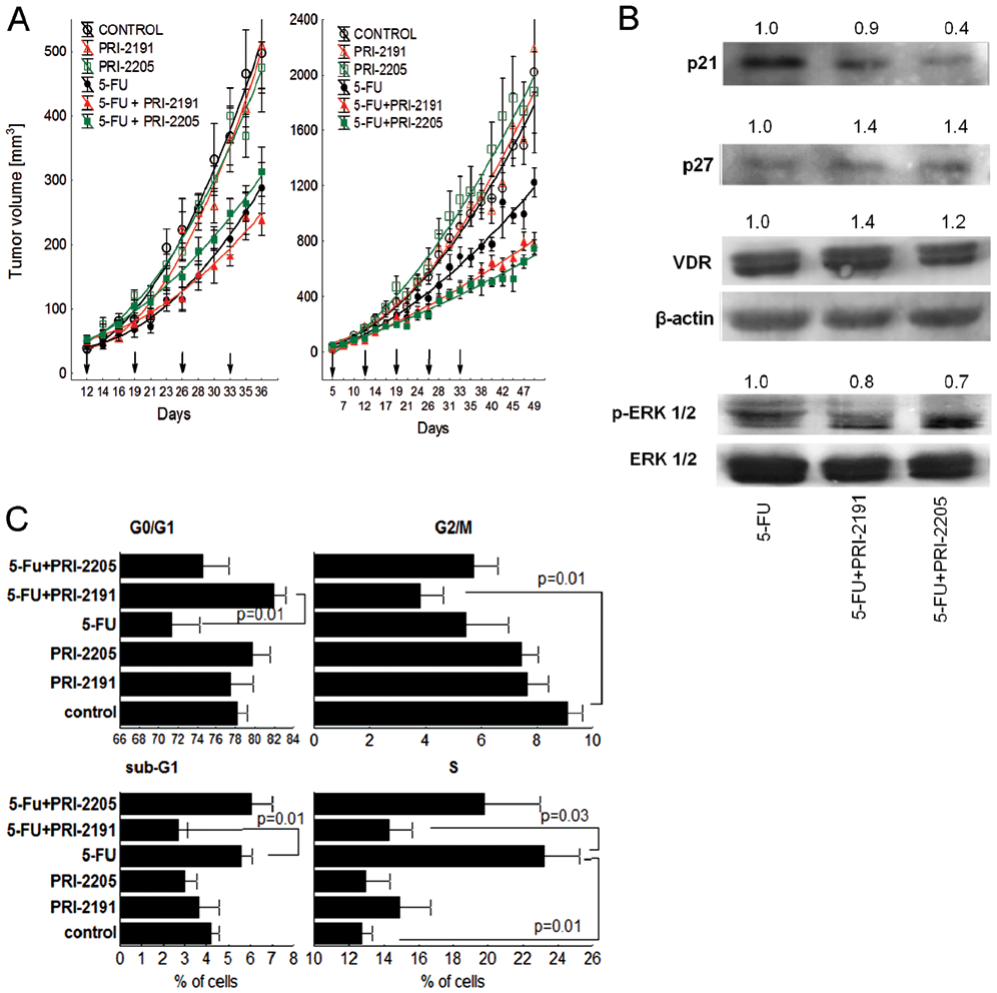
HT-29 tumor growth in mice treated with a combination of 5-FU and vitamin D analogs. (A) Left graph, administration of vitamin D analogs five times a week; right graph, administration of vitamin D analogs three times a week. (B) Western blot analysis of tumor tissue (tumors from mice treated three times a week). Specimens of the tumor tissue from euthanized animals were collected in liquid nitrogen and stored at −80°C; then, they were mechanically homogenized. Equal amounts of protein (25 μg for detecting VDR; 100 μg for p21, p27, ERK and p-ERK and 25 μg for β-actin) were used. Densitometric analysis of the western blots was carried out using ImageJ 1.46r. Blots were normalized to actin or ERK and the fold-change protein level expression is reported in comparison to control. (C) Tumor cell cycle analysis (tumors from mice treated five times a week). Analysis was performed using ModFit LT 3.0 software. Statistical analysis, Kruskal-Wallis multiple comparison test. Number of mice, (A and C) 8 per group, (B) 7–8 per group. 5-FU, 5-fluorouracil.

In combined treatment using the first protocol (analogs administered 5 times a week), analog PRI-2205 did not influence tumor growth. From day 28 of the experiment, analog PRI-2191 showed a tendency to reduce tumor volume in combination with 5-FU and its interaction could be explained as synergism (Fig. 4A, left graph).

The second treatment schedule was considerably more effective. Both agents potentiate the antitumor activity of 5-FU. A statistically significant retardation of tumor growth was observed in mice treated with 5-FU and PRI-2191 from day 28 to the end of the experiment (with the exception of days 40 and 47). PRI-2205 used with 5-FU significantly retarded tumor growth from day 24 to the end of measurements. Synergy was observed on all measurement days (Fig. 4A, right graph).

#### Cell cycle analysis (analogs administered 5 times a week)

Cell cycle analysis was performed on tumors harvested from mice treated both with 5-FU alone and combined with both analogs. It was observed that, in tumors treated with 5-FU, the percentage of cells in the G_0_/G_1_ stage significantly decreased. Moreover, these cells were stopped in the S phase (P<0.05) and the percentage of cell deaths increased. PRI-2191 used alone did not significantly affect the cell cycle of HT-29 tumors. However, used in combined treatment with 5-FU, it increased the number of cells in G_0_/G_1_ and in parallel decreased the cells in both the G_2_/M and S stages as well as death cells as compared to 5-FU alone (Fig. 4C). Tumors from mice treated with PRI-2205 combined with 5-FU showed a similar cell cycle distribution to that of mice treated with 5-FU alone (Fig. 4C).

#### Western blot analysis of selected groups of mice (analogs administered 5 times a week)

For this analysis tumors from mice treated with 5-FU alone or combined with vitamin D analogs were harvested. The results of the western blot studies showed that, in tumors from mice treated with 5-FU combined with PRI-2191 or PRI-2205, the expression of p27 and VDR was increased as compared to tumors from animals treated with 5-FU alone. The expression of p21, as well as p-ERK1/2 was diminished when mice were administered with 5-FU combined with both analogs. This diminution was well-defined for PRI-2205 (Fig. 4B).

### Effect of PRI-2191 or PRI-2205 administered alone or in combination with 5-FU on mice bearing orthotopic human colon cancer HT-29

#### Primary tumor growth

In these experiments, we used the optimal treatment schedule to assess the antitumor activity of combined treatment against tumors growing intra-intestinally.

**Figure 5:**
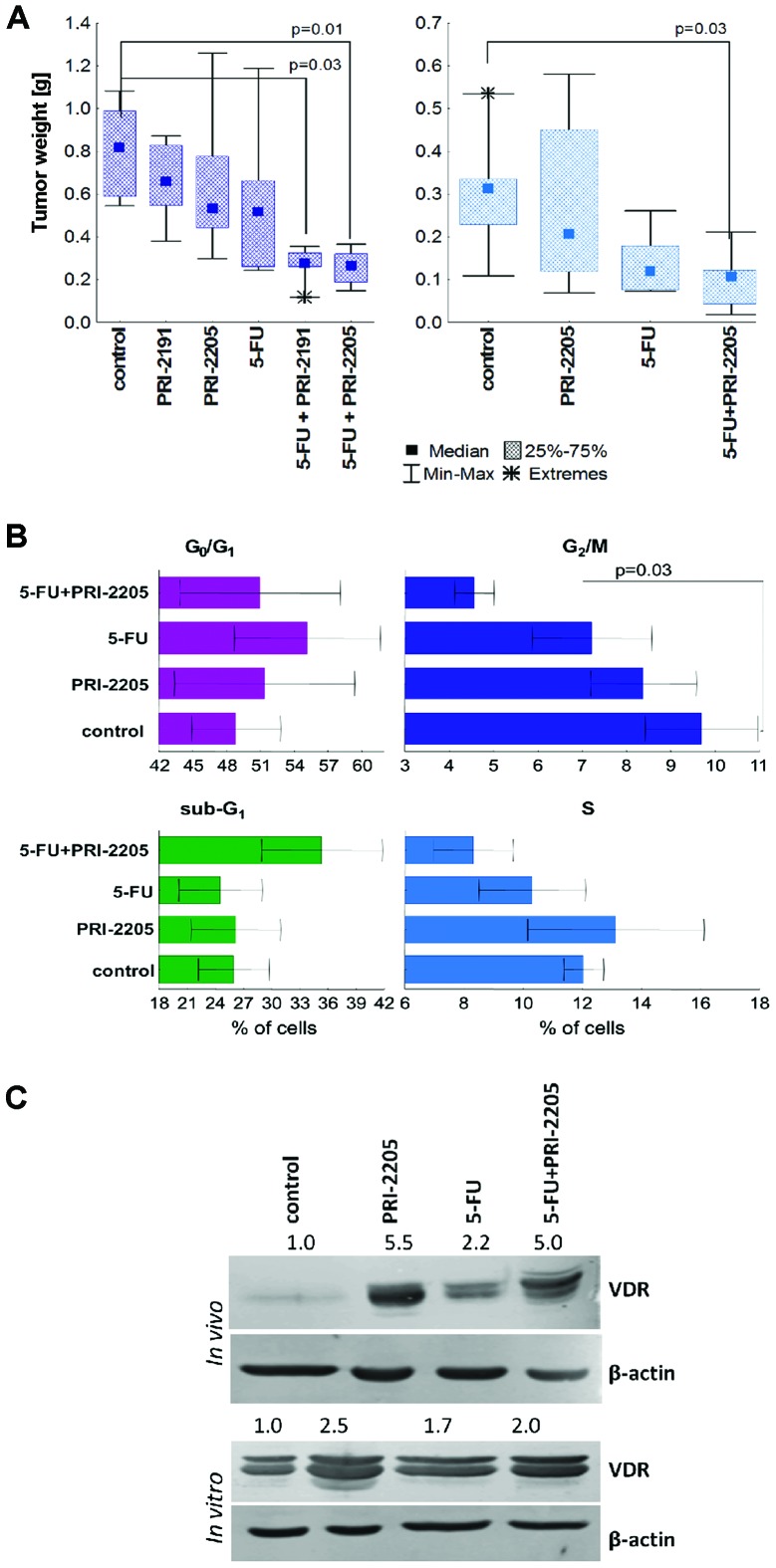
Intestinal tumor growth inhibition by treatment with 5-FU combined with vitamin D analogs. (A) Left graph, intravenous administration of 5-FU; right graph, intraperitoneal administration of 5-FU. (B) Tumor cell cycle analysis. (C) VDR expression in intestinal tumors and HT-29 cells from *in vitro* culture. For cell cycle distribution and VDR expression, cells were prepared from HT-29 tumors grown in the cecal wall of mice, as described in Materials and methods. Cell cycle distribution-FACS analysis, the results are presented as a mean (± SD) percentage of the cell population attributed to one of the cell cycle phases, G_0_/G_1_, S, G_2_/M and sub-G_1_. Statistical analysis using Kruskall Wallis multiple comparison test. N=5 in each group. VDR expression; cells from tumors (upper panel) or from *in vitro* culture (lower panel). Equal amounts of protein (25 μg of cultured cells or 50 μg of tumors for detecting VDR or β-actin) were used. Densitometric analysis of the western blots was carried out using ImageJ 1.46r. Blots were normalized to actin and the fold-change protein level expression is reported in comparison to control. 5-FU, 5-fluorouracil.

Analyzing the weight of intestinal tumors harvested from mice, we observed a statistically significant reduction in tumor growth in mice treated with 5-FU and both analogs (Fig. 5A). In the left-hand graph, the results of experiments with an i.v. route of injection of 5-FU are shown. 5-FU alone reduced tumor weight by 37% as compared to the control, whereas used with PRI-2191 or PRI-2205 the reduction was 66 and 68%, respectively (Table II) and this effect was synergistic. Similar results were observed after i.p. administration of 5-FU with PRI-2205 (Fig. 5A, right graph). The schedules of treatment used did not affect the body weight of animals (data not shown). Moreover, when the serum calcium level was analyzed, we did not observe any significant changes (Table II). The blood leukocyte count had decreased by 5-FU and a further decrease was observed in combined treatment with PRI-2205 (Table II). This analysis was not performed for PRI-2191.

#### Cell cycle analysis and VDR expression (i.p. administration of 5-FU)

Analyzing cell cycle and cell death in cells derived from HT-29 intestinal tumors, we showed a significant decrease in the percentage of cells in the G_2_M phase and a tendency to increase the number of cell deaths in tumors from mice treated with 5-FU and PRI-2205 simultaneously (Fig. 5B). The expression of VDR increased in treated groups as compared to tumors from control mice. A 5 fold-increase in VDR expression as compared to control tumors was observed in tumors from mice treated with both agents and with PRI-2205 alone (Fig. 5C). We observed a similar tendency in HT-29 cells from *in vitro* culture (Fig. 5C).

### Effect of PRI-2191 or PRI-2205 administered either alone or in combination with CPC on mice bearing subcutaneous human colon cancer HT-29

CPC, in the doses and schedules used, did not influence tumor growth until day 38 of the experiment, when some retardation was observed. In this model, neither vitamin D analog improved the anticancer activity of CPC (data not shown).

### Molecular modeling

**Figure 6:**
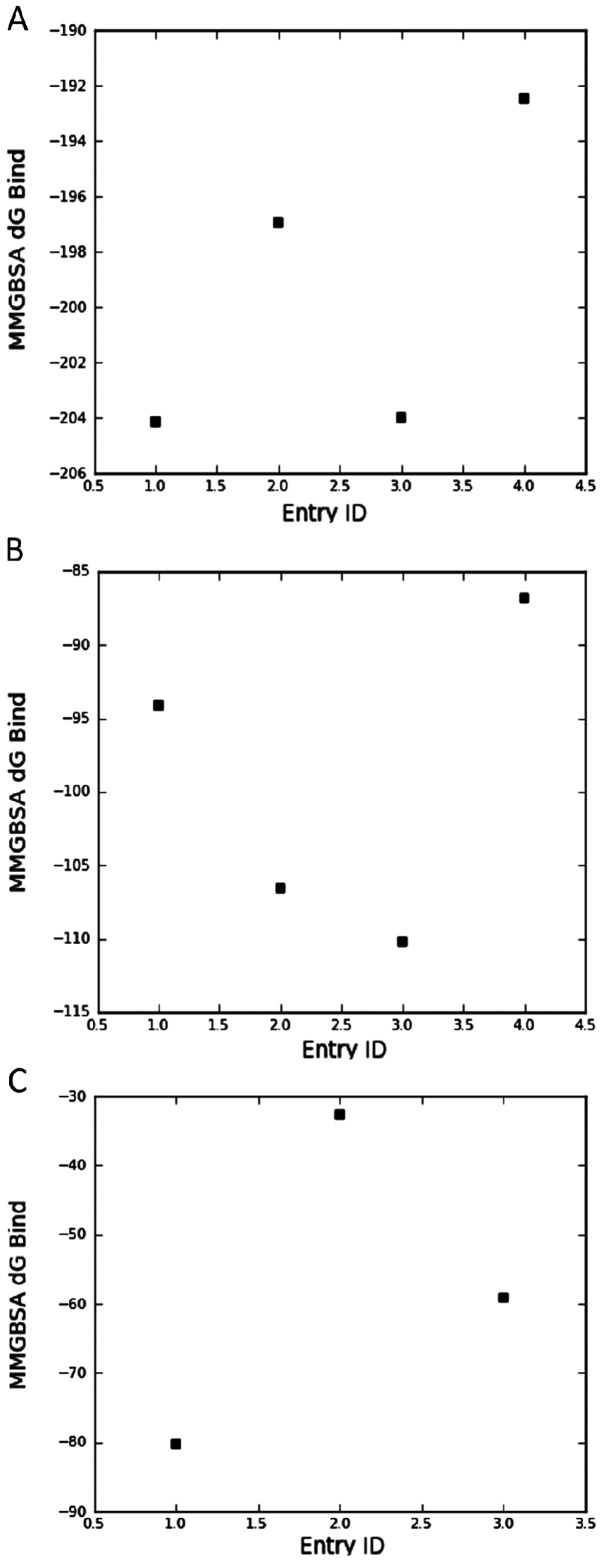
Estimate of vitamin D analogs binding energy to vitamin D receptor (A), vitamin D-binding protein (B) and constitutive androstane receptor (C). Entry ID: 1, calcipotriol; 2, PRI-2191; 3, PRI-2205; and 4, 1,25(OH)_2_D_3_. Binding energy was estimated in kcal/mol. Analogs were docked into the space occupied by the original ligand and the binding energy was estimated using an implicit solvation model as described in Materials and methods. (C) 1,25(OH)_2_D_3_ did not fit into the pocket and was not included.

The vitamin D compounds 1,25(OH)_2_D_3_, PRI-2191, PRI-2205 and calcipotriol were docked into the space occupied by the original ligand [1,25(OH)_2_D_3_; 25(OH)D_3_; 3,20-pregnanedione for VDR, DBP and CAR, respectively] and the binding energy was estimated using an implicit solvation model. The results presented in Fig. 6A show that the estimated binding energy to VDR calculated for PRI-2205 is similar to its parent compound, namely calcipotriol, and is higher than that observed for PRI-2191 and 1,25(OH)_2_D_3_. On the other hand, analyzing binding energy to DBP (Fig. 6B), we conclude that both analogs tested, PRI-2191 and PRI-2205, have higher affinity to DBP than the parent compounds, 1,25(OH)_2_D_3_ and calcipotriol.

1,25(OH)_2_D_3_ did not interact with the ligand binding domain of CAR/RXR protein complex. However, PRI-2205 possesses a reasonable affinity to this receptor which was higher than for tacalcitol (Fig. 6C). The affinity of PRI-2201 was the highest from the three mentioned compounds.

## Discussion

1,25(OH)_2_D_3_ (calcitriol) and its analogs are known to be agents which exert anticancer activities both alone and by cooperating with anticancer compounds (17,48). In our previous experiments with mouse colon cancer MC38 *in vivo*, we showed that 1,25(OH)_2_D_3_ analogs interact synergistically with 5-FU improving its antitumor and antimetastatic activity, as well as prolonging the survival time of mice. However, better results were observed for PRI-2191 than for PRI-2205 (49). In the present study, we analyzed the antitumor effect of such combined treatment on immunodeficient mice bearing human HT-29 colon cancer. We showed that the three times a week schedule of treatment is more effective than the other schedule. However, analog PRI-2191 in combined treatment with 5-FU retarded subcutaneous tumor growth in both schedules (three and five times a week of administration of vitamin D analogs). The improved antitumor effect of 5-FU by PRI-2191 or PRI-2205 was also observed in mice bearing human colon cancer HT-29 transplanted orthotopically.

It has been previously shown that proliferating HT-29 colon cancer cells exhibit upregulation of VDR and induction of 24-hydroxylase mRNA, whereas differentiated cells fail to exhibit either of these biological responses to 1,25(OH)_2_D_3_ (50). Moreover, differentiation of colon cancer cells induced by various treatments occurs via upregulation of VDR (50,51). Palmer *et al* reported that vitamin D analogs promote differentiation only of colon cancer cells expressing VDR and that this process is related to induction of E-cadherin and inhibition of β-catenin signaling (52). The analysis of VDR expression in orthotopic or subcutaneous tumors from mice treated with 5-FU and PRI-2205 showed either a 2.3- or only a 1.2-fold increase as compared to 5-FU, respectively. In subcutaneous tumors, PRI-2191 also slightly increased (1.4-fold) expression of VDR as compared to 5-FU alone. In our previous studies, we observed a 6-fold increase in VDR expression in subcutaneous HT-29 tumors by PRI-2191 used alone, but not by PRI-2205 (30). The differences between the significant increase (5.5-fold) in VDR in orthotopic tumors and that observed subcutaneously (0.8-fold) by PRI-2205 (30) could be explained via the importance of the tumor microenvironment in which cancer cells are implanted (53,54). HT-29 cells incubated with these agents in *in vitro* culture showed a similar pattern of VDR expression to that of orthotopic tumors (Fig. 5C).

The ligand binding domain (LBD) of the VDR protein has been well characterized using the known amino acid sequence of the VDR and its crystallized structure. An evaluation of how 1,25(OH)_2_D_3_ is oriented within the LBD has provided important insight into the chemical interactions that are responsible for ligand-receptor binding. These interactions have been essential for the elucidation of the structure function relationship between the VDR, 1,25(OH)_2_D_3_ and the development of new 1,25(OH)_2_D_3_ analogs (55,56). It is known that PRI-2191 [tacalcitol, 1,24-(OH)_2_D_3_] has a similar or higher affinity for the VDR than the parent compound 1,25(OH)_2_D_3_ (57). On the other hand, the relative affinities to the VDR of some analogs with high anticancer activity, such as KH1060, calcipotriol (MC-903) and EB 1089, were similar or lower compared to 1,25(OH)_2_D_3_ (55). The estimated energy of binding PRI-2205 to LBD of VDR as calculated in our studies was higher than that for PRI-2191; moreover, both analogs bind to ligand binding pocket of vitamin D binding protein (DBP) with similar affinity. The biological activity of vitamin D metabolites and analogs depends on their affinity not only for the VDR but also for vitamin D binding protein. One of the functions of DBP is to prolong the lifetime of 1,25(OH)_2_D_3_ in circulation. Analogs with a low DBP but good receptor binding properties display low *in vivo* biologic activity on calcium and bone homeostasis, at least partly due to the altered pharmacokinetics (58). However, the biological activity *in vivo* of secosteroids could also be modulated through its potential interaction with constitutive androstane receptor (CAR) and a crosstalk CAR-VDR. Constitutive androstane receptor was originally identified as a xenobiotic sensor that regulates the expression of cytochrome P450 genes. However, previous studies suggest that this nuclear receptor is also involved in the regulation of energy metabolism including glucose and lipid homeostasis (59,60). One of the VDR target genes is CYP24 which encodes a mitochondrial cytochrome P450 that hydroxylates 1,25(OH)_2_D_3_ and other vitamin D derivatives at position 24. This biotransformation converts these molecules to inactive metabolites. Transactivation of two responsive elements (VDRE-1 and VDRE-2) in CYP24 by VDR/RXR leads to increased expression of CYP24, reduced level of the biologically active 1,25(OH)_2_D_3_ and downregulation of VDR-target genes (61). Notably, Moreou *et al* (62) showed that CAR binds to and transactivates the VDREs present in the CYP24 promoter and that a specific human CAR agonist, CITCO, increases CYP24 mRNA expression in primary human hepatocytes. These findings suggest that the VDR-CAR crosstalk resulting from the recognition of same response elements is reciprocal (62). Such crosstalk provides, at least in part, an objective explanation to the observation from our studies indicating similar biological activity of PRI-2191 and PRI-2205, but lower toxicity of the latter (26). As we showed, PRI-2205 has stronger affinity for CAR/RXR complex than PRI-2191 and in parallel possesses higher binding affinity for VDR LBD. This characteristic of PRI-2205 may be the possible explanation of its low toxicity via increased generation of CYP24 through VDR and CAR activation, with a sustained, but lower, anticancer activity through the requirement for higher doses needed to obtain results similar to PRI-2191. The last conclusion is also supported by our docking studies showing a high affinity to VDR as well as the best binding to DBP.

Cell cycle distribution in cells from *in vitro* culture as well as from tumors was not influenced by PRI-2205 used alone. However, we observed a similar general tendency in all experimental conditions towards a decrease in the number of cells in G_0_/G_1_, G_2_M and S as compared to 5-FU alone. Moreover, in orthotopic tumors as well as in cultured cells, an increase in cell deaths was observed in combined treatment regimens. PRI-2191 acted differently, increasing the percentage of cells in G_0_/G_1_ and decreasing it in S and G_2_/M as compared to 5-FU alone. This suggests the pro-differentiating activity of this analog, particularly when we take into consideration our previous results showing that PRI-2191 increased the expression of E-cadherin on HT-29 cells *in vitro* (49). There, it was shown that the target gene of p53 - p21^waf1/cip1^ is a primary 1,25(OH)_2_D_3_-responding gene with VDR binding promoter regions (10). Moreover, other vitamin D targeted genes which lack VDRE, such as the other cell cycle inhibitor p27, could be regulated by 1,25(OH)_2_D_3_ (63,64). Several studies suggest that growth inhibition by 1,25(OH)_2_D_3_ may be attributed to inhibition of the G_1_ to S cell cycle transition, which may be due, at least in part, to stimulation of the expression of the cyclin-dependent kinase inhibitors (CDKIs), p21 and p27, as well as to programmed cell death (10). Our present observations are in accordance with previous results on the pro-differentiating activity of PRI-2191. We have shown that HL-60 leukemia cells acquire a mature macrophage phenotype after their exposure to PRI-2191 *in vitro* (21). Moreover, we also observed that PRI-2191 increased the expression of p27 in mouse mammary gland tumors (29). These data suggest that the higher antitumor effect of the combined treatment with PRI-2191 and 5-FU may be the result of the cell cycle arrest in the G_0_/G_1_ cell cycle phase and the increased p21 and p27 expression following treatment with PRI-2191. However, the lack of increase in the p27 expression following treatment with PRI-2205 suggests that there may be differences between the mechanisms of action of these two analogs.

1,25(OH)_2_D_3_ or its analogs could reveal various activities, balanced between pro- and anti-apoptotic pathways. In particular, 1,25(OH)_2_D_3_ increased the level of pro-apoptotic protein BAK (BCL-2 family member) (65) and decreased the anti-apoptotic activity of β-catenin (52). Analysis of the cell death parameters in *in vitro* culture suggested antagonism in pro-apoptotic activity, especially of PRI-2191 and 5-FU. The percentage of PI stained cells, active caspase-3 as well as p53 positive cells decreased in comparison to 5-FU. PRI-2191 also reduced the number of both cells with lower mitochondrial potential and Annexin V positive cells. A similar effect was described after simultaneous incubation of HT-29 cells with H_2_O_2_ and 1,25(OH)_2_D_3_. The increased cytotoxic effect of simultaneous treatment was demonstrated, but the activity of caspase 3 was decreased by 1,25(OH)_2_D_3_ as compared to H_2_O_2_ (66). PRI-2205 slightly decreased only PI positive cells, but did not affect caspase-3 or p-53 positive cells as compared to 5-FU alone. HT-29 possessed mutated p53 (67) and, according to recent studies, a functional and physical interaction between mutated p53 and the vitamin D transcriptional regulatory pathway exists. This interaction lies in the cooperation between mutated p53 with 1,25(OH)_2_D_3_ to elicit an anti-apoptotic state (68). Since PRI-2191 possesses a similar pattern of activity to 1,25(OH)_2_D_3_, the presence of mutated p53 in HT-29 cells may be the reason of the observed anti-apoptotic effects. However, in spite of these disadvantageous effects regarding apoptosis, the therapeutic effect expressed as tumor growth retardation is significant. In contrast, PRI-2205, several activities of which differ from those observed for 1,25(OH)_2_D_3_ or PRI-2191 (such as no influence on the expression of p53), did not reveal anti-apoptotic activity. Moreover, our previous studies, including on HL-60 leukemia cell lines, showed the pro-apoptotic activity of this analog (26).

5-FU is an important drug used in the chemotherapy of colorectal cancer, but new strategies for combined treatment with this agent are still under development. It has been assumed that the low thymidylate synthase (TS; a direct target of 5-FU) level increases drug efficacy (69). Previous data as well as our own results showed that HT-29 cells (with mutated TP53) exposed to 5-FU are cumulated in S cell cycle phase which is correlated with high TS level during DNA synthesis (70). High level of TS leads to insensitivity of tumor cells to 5-FU and is one of the main reasons of resistance development (71). Takagi *et al* (72) showed that the HCT116 colon cancer cells with silenced expression of p21 protein express higher level of TS than wild-type cells. Both analogs induced the expression CDKIs p21 and/or p27 and could probably block the expression of TS and therefore increase the 5-FU activity. Some agents are known as sensitizing cells to 5-FU via various mechanisms. An example is trametinib, which induced p15 and p27 expression and reduced cyclin D1 levels. Another one is fenofibrate which dephosphorylated ERK1/2 and reduced cyclin D1 levels in HT-29 cells with subsequent G_1_-phase arrest, reduced TS expression and sensitized cells to 5-FU (73). PRI-2191 and PRI-2205 possess a similar profile of activity, also reducing the level of p-ERK1/2 as compared to 5-FU alone.

In conclusion, our results suggest that the mechanism of potentiation of 5-FU antitumor action by both analogs is realized via increased p21 expression and decreased phosphorylation level of ERK1/2 which may lead to diminution of thymidylate synthase expression. However, the differences in the biological activity including mode of action and toxicity between both analogs was observed. Our molecular modeling studies may in part clarify some of these differences, namely, in general PRI-2205 possesses higher binding affinity for VDR, DBP, but also for CAR\RXR ligand binding domain which may, in part, explain its very low toxicity with sustained anticancer activity. However, the combined treatment proposed in our studies was ineffective in the HT-29 colon cancer model, when capecitabine was used in place of 5-FU.

## Figures and Tables

**Table I:** The proliferation inhibition and cell cycle distribution of HT-29 colon cancer cells *in vitro*.

Compounds	Proliferation inhibition (%)	Cell cycle distribution (% of cells)			

		G_0_/G_1_	S	G_2_/M	Sub-G_1_
Control	-	49.9±4.9	38.6±4.2	11.5±1.7	0.4±0.5
EtOH	1.5±1.5	49.2±4.9	37.6±5.8	13.2±1.3	0.7±0.3
1,25(OH)_2_D_3_	7.1±3.9	56.1±3.7	32.4±4.4	11.5±1.6	0.1±0.1
PRI-2191	1.6±3.0	59.0±1.4	30.4±1.1	10.6±0.7	0.1±0.2
Calcipotriol	3.2±3.1	58.5±0.4	31.8±1.7	9.7±2.3	0.1±0.2
PRI-2205	4.6±1.8	51.2±4.6	36.7±4.6	12.2±2.2	0.9±0.8
5-FU	49.9±4.6	41.5±7.4	33.2±8.7	25.3±15.2	0.1±0.2
5-FU+Calcitriol	54.1±4.4	40.1±1.6	21.2±0.8	38.8±0.8	0.2±0.3
5-FU+PRI-2191	52.3±5.1	39.1±2.0	21.3±2.3^a^	39.6±2.4	0.1±0.1
5-FU+PRI-2201	50.9±4.4	38.6±0.5	20.6±1.5^a^	40.9±1.1	0
5-FU+PRI-2205	53.0±3.9	49.6±3.7	36.9±4.0	13.8±0.7	6.9±7.8

HT-29 colon cancer cells were incubated with vitamin D analogs (100 nM) and 5-FU (200 μg/ml) for 48 h. Cell cycle distribution-FACS analysis; the results are presented as a mean (± SD) percentage of the cell population attributed to one of the cell cycle phases: G_0_/G_1_, S, G_2_/M and sub-G_1_. Analysis was performed using ModFit LT 3.0 software.

a P<0.05.

**Table II:** The intestinal tumor growth, calcium level and blood leukocyte count in mice bearing HT-29 tumors.

	5-FU intraperitoneally					5-FU intravenously				
		
Group	Tumor weight on day 39 (g)	TGI (%)	H (%)	Leukocytes (thousands/μl)	N	Tumor weight on day 53 (g)	TGI (%)	H (%)	Calcium (mmol/l)	N
Control	0.305±0.140			5.5±1.3	6	0.808±0.24	--		2.50±0.07	6
PRI-2191	nt.	nt.		nt.		0.657±0.20	20		2.61±0.07	5
PRI-2205	0.291±0.199	5		5.7±0.6	7	0.663±0.38	35		2.42±0.08	5
5-FU	0.138±0.072	55		4.2±1.3	6	0.527±0.33	37		2.48±0.03	7
5-FU + PRI-2191	nt.	nt.		nt.		0.268±0.09^a^	66	49	2.55±0.04	
5-FU + PRI-2205	0.097±0.062^a^	68	57	3.5±1.0	7	0.259±0.08^a^	68	59	2.49±0.02	6

(%) H, the expected inhibition used to estimate the effect of the combination of two compounds was evaluated using the formula (%) H = 100 − [(100 − E for cytostatic) × (100 − E for calcitriol analog)/100], where E was tumor growth inhibition (TGI). N, no. of mice/group; nt., not tested.

a P<0.05 as compared to control and PRI-2205, ANOVA followed by Tukey HSD test for unequal N.

## References

[1] Lamprecht SA, Lipkin M (2003). Chemoprevention of colon cancer by calcium, vitamin D and folate: molecular mechanisms. Nat Rev Cancer.

[2] McCarthy TC, Li X, Sinal CJ (2005). Vitamin D receptor-dependent regulation of colon multidrug resistance-associated protein 3 gene expression by bile acids. J Biol Chem.

[3] Pritchard RS, Baron JA, Gerhardsson de Verdier M (1996). Dietary calcium, vitamin D, and the risk of colorectal cancer in Stockholm, Sweden. Cancer Epidemiol Biomarkers Prev.

[4] Terry P, Baron JA, Bergkvist L, Holmberg L, Wolk A (2002). Dietary calcium and vitamin D intake and risk of colorectal cancer: a prospective cohort study in women. Nutr Cancer.

[5] Berkovich L, Sintov AC, Ben-Shabat S (2013). Inhibition of cancer growth and induction of apoptosis by BGP-13 and BGP-15, new calcipotriene-derived vitamin D_3_analogs, in-vitro and in-vivo studies. Invest New Drugs.

[6] Grau MV, Baron JA, Sandler RS, Haile RW, Beach ML, Church TR, Heber D (2003). Vitamin D, calcium supplementation, and colorectal adenomas: results of a randomized trial. J Natl Cancer Inst.

[7] Hartman TJ, Albert PS, Snyder K, Slattery ML, Caan B, Paskett E, Iber F, Kikendall JW, Marshall J, Shike M, Weissfeld J, Brewer B, Schatzkin A, Lanza E (2005). The association of calcium and vitamin D with risk of colorectal adenomas. J Nutr.

[8] Baniahmad A, Tsai MJ (1993). Mechanisms of transcriptional activation by steroid hormone receptors. J Cell Biochem.

[9] Nagpal S, Na S, Rathnachalam R (2005). Noncalcemic actions of vitamin D receptor ligands. Endocr Rev.

[10] Saramaki A, Banwell CM, Campbell MJ, Carlberg C (2006). Regulation of the human p21 (waf1/cip1) gene promoter via multiple binding sites for p53 and the vitamin D_3_receptor. Nucleic Acids Res.

[11] Alvarez-Diaz S, Valle N, Ferrer-Mayorga G, Lombardia L, Herrera M, Dominguez O, Segura MF, Bonilla F, Hernando E, Munoz A (2012). MicroRNA-22 is induced by vitamin D and contributes to its antiproliferative, antimigratory and gene regulatory effects in colon cancer cells. Hum Mol Genet.

[12] Matusiak D, Murillo G, Carroll RE, Mehta RG, Benya RV (2005). Expression of vitamin D receptor and 25-hydroxyvitamin D3-1{alpha}-hydroxylase in normal and malignant human colon. Cancer Epidemiol Biomarkers Prev.

[13] Tangpricha V, Spina C, Yao M, Chen TC, Wolfe MM, Holick MF (2005). Vitamin D deficiency enhances the growth of MC-26 colon cancer xenografts in Balb/c mice. J Nutr.

[14] Mokady E, Schwartz B, Shany S, Lamprecht SA (2000). A protective role of dietary vitamin D_3_in rat colon carcinogenesis. Nutr Cancer.

[15] Hummel DM, Thiem U, Hobaus J, Mesteri I, Gober L, Stremnitzer C, Graca J, Obermayer-Pietsch B, Kallay E (2013). Prevention of preneoplastic lesions by dietary vitamin D in a mouse model of colorectal carcinogenesis. J Steroid Biochem Mol Biol.

[16] Larriba MJ, Ordonez-Moran P, Chicote I, Martin-Fernandez G, Puig I, Munoz A, Palmer HG (2011). Vitamin D receptor deficiency enhances Wnt/β-catenin signaling and tumor burden in colon cancer. PLoS ONE.

[17] Cho YL, Christensen C, Saunders DE, Lawrence WD, Deppe G, Malviya VK, Malone JM (1991). Combined effects of 1,25-dihydroxyvitamin D_3_ and platinum drugs on the growth of MCF-7 cells. Cancer Res.

[18] Ravid A, Rocker D, Machlenkin A, Rotem C, Hochman A, Kessler-Icekson G, Liberman UA, Koren R (1999). 1,25-Dihydroxyvitamin D3 enhances the susceptibility of breast cancer cells to doxorubicin-induced oxidative damage. Cancer Res.

[19] Siwinska A, Opolski A, Chrobak A, Wietrzyk J, Wojdat E, Kutner A, Szelejewski W, Radzikowski C (2001). Potentiation of the antiproliferative effect in vitro of doxorubicin, cisplatin and genistein by new analogues of vitamin D. Anticancer Res.

[20] Pelczynska M, Switalska M, Maciejewska M, Jaroszewicz I, Kutner A, Opolski A (2006). Antiproliferative activity of vitamin D compounds in combination with cytostatics. Anticancer Res.

[21] Opolski A, Wietrzyk J, Siwinska A, Marcinkowska E, Chrobak A, Radzikowski C, Kutner A (2000). Biological activity in vitro of side-chain modified analogues of calcitriol. Curr Pharm Des.

[22] Kota BP, Allen JD, Roufogalis BD (2011). The effect of vitamin D_3_and ketoconazole combination on VDR-mediated P-gp expression and function in human colon adenocarcinoma cells: implications in drug disposition and resistance. Basic Clin Pharmacol Toxicol.

[23] Abe J, Nakano T, Nishii Y, Matsumoto T, Ogata E, Ikeda K (1991). A novel vitamin D_3_analog, 22-oxa-1,25-dihydroxyvitamin D3, inhibits the growth of human breast cancer in vitro and in vivo without causing hypercalcemia. Endocrinology.

[24] Abe-Hashimoto J, Kikuchi T, Matsumoto T, Nishii Y, Ogata E, Ikeda K (1993). Antitumor effect of 22-oxa-calcitriol, a noncalcemic analogue of calcitriol, in athymic mice implanted with human breast carcinoma and its synergism with tamoxifen. Cancer Res.

[25] Chodyński M, Wietrzyk J, Marcinkowska E, Opolski A, Szelejewski W, Kutner A (2002). Synthesis and antiproliferative activity of side-chain unsaturated and homologated analogs of 1,25-dihydroxyvitamin D(2). (24E)-(1S)-24-Dehydro-24a-homo-1,25-dihydroxyergocalciferol and congeners. Steroids.

[26] Wietrzyk J, Chodynski M, Fitak H, Wojdat E, Kutner A, Opolski A (2007). Antitumor properties of diastereomeric and geometric analogs of vitamin D_3_. Anticancer Drugs.

[27] Wietrzyk J, Nevozhay D, Filip B, Milczarek M, Kutner A (2007). The antitumor effect of lowered doses of cytostatics combined with new analogs of vitamin D in mice. Anticancer Res.

[28] Wietrzyk J, Pelczynska M, Madej J, Dzimira S, Kusnierczyk H, Kutner A, Szelejewski W, Opolski A (2004). Toxicity and antineoplastic effect of (24R)-1,24-dihydroxyvitamin D3 (PRI-2191). Steroids.

[29] Wietrzyk J, Nevozhay D, Milczarek M, Filip B, Kutner A (2008). Toxicity and antitumor activity of the vitamin D analogs PRI-1906 and PRI-1907 in combined treatment with cyclophosphamide in a mouse mammary cancer model. Cancer Chemother Pharmacol.

[30] Milczarek M, Rosinska S, Psurski M, Maciejewska M, Kutner A, Wietrzyk J (2013). Combined colonic cancer treatment with vitamin D analogs and irinotecan or oxaliplatin. Anticancer Res.

[31] Lowe SW, Bodis S, McClatchey A, Remington L, Ruley HE, Fisher DE, Housman DE, Jacks T (1994). p53 status and the efficacy of cancer therapy in vivo. Science.

[32] Sun XX, Dai MS, Lu H (2007). 5-fluorouracil activation of p53 involves an MDM2-ribosomal protein interaction. J Biol Chem.

[33] Chakrabarty S, Radjendirane V, Appelman H, Varani J (2003). Extracellular calcium and calcium sensing receptor function in human colon carcinomas: promotion of E-cadherin expression and suppression of beta-catenin/TCF activation. Cancer Res.

[34] Bhagavathula N, Hanosh AW, Nerusu KC, Appelman H, Chakrabarty S, Varani J (2007). Regulation of E-cadherin and beta-catenin by Ca^2+^in colon carcinoma is dependent on calcium-sensing receptor expression and function. Int J Cancer.

[35] Chakrabarty S, Wang H, Canaff L, Hendy GN, Appelman H, Varani J (2005). Calcium sensing receptor in human colon carcinoma: interaction with Ca^2+^and 1,25-dihydroxyvitamin D_3_. Cancer Res.

[36] Wang X, Chen W, Singh N, Promkan M, Liu G (2010). Effects of potential calcium sensing receptor inducers on promoting chemosensitivity of human colon carcinoma cells. Int J Oncol.

[37] Liu G, Hu X, Chakrabarty S (2010). Vitamin D mediates its action in human colon carcinoma cells in a calcium-sensing receptor-dependent manner: downregulates malignant cell behavior and the expression of thymidylate synthase and survivin and promotes cellular sensitivity to 5-FU. Int J Cancer.

[38] Peters GJ, van der Wilt CL, van Moorsel CJ, Kroep JR, Bergman AM, Ackland SP (2000). Basis for effective combination cancer chemotherapy with antimetabolites. Pharmacol Ther.

[39] Kim R, Emi M, Tanabe K, Uchida Y, Arihiro K (2006). The role of apoptotic or nonapoptotic cell death in determining cellular response to anticancer treatment. Eur J Surg Oncol.

[40] Sastry GM, Adzhigirey M, Day T, Annabhimoju R, Sherman W (2013). Protein and ligand preparation: parameters, protocols, and influence on virtual screening enrichments. J Comput Aided Mol Des.

[41] Friesner RA, Murphy RB, Repasky MP, Frye LL, Greenwood JR, Halgren TA, Sanschagrin PC, Mainz DT (2006). Extra precision glide: docking and scoring incorporating a model of hydrophobic enclosure for protein-ligand complexes. J Med Chem.

[42] Halgren TA, Murphy RB, Friesner RA, Beard HS, Frye LL, Pollard WT, Banks JL (2004). Glide: a new approach for rapid, accurate docking and scoring. 2. Enrichment factors in database screening. J Med Chem.

[43] Friesner RA, Banks JL, Murphy RB, Halgren TA, Klicic JJ, Mainz DT, Repasky MP, Knoll EH, Shelley M, Perry JK, Shaw DE, Francis P, Shenkin PS (2004). Glide: a new approach for rapid, accurate docking and scoring. 1. Method and assessment of docking accuracy. J Med Chem.

[44] Li J, Abel R, Zhu K, Cao Y, Zhao S, Friesner RA (2011). The VSGB 2.0 model: a next generation energy model for high resolution protein structure modeling. Proteins.

[45] Hou T, Wang J, Li Y, Wang W (2011). Assessing the performance of the MM/PBSA and MM/GBSA methods. 1. The accuracy of binding free energy calculations based on molecular dynamics simulations. J Chem Inf Model.

[46] Hou T, Wang J, Li Y, Wang W (2011). Assessing the performance of the molecular mechanics/Poisson Boltzmann surface area and molecular mechanics/generalized Born surface area methods. II. The accuracy of ranking poses generated from docking. J Comput Chem.

[47] Rastelli G, Del Rio A, Degliesposti G, Sgobba M (2010). Fast and accurate predictions of binding free energies using MM-PBSA and MM-GBSA. J Comput Chem.

[48] Danilenko M, Studzinski GP (2004). Enhancement by other compounds of the anti-cancer activity of vitamin D_3_and its analogs. Exp Cell Res.

[49] Milczarek M, Psurski M, Kutner A, Wietrzyk J (2013). Vitamin D analogs enhance the anticancer activity of 5-fluorouracil in an in vivo mouse colon cancer model. BMC Cancer.

[50] Zhao X, Feldman D (1993). Regulation of vitamin D receptor abundance and responsiveness during differentiation of HT-29 human colon cancer cells. Endocrinology.

[51] Gaschott T, Werz O, Steinmeyer A, Steinhilber D, Stein J (2001). Butyrate-induced differentiation of Caco-2 cells is mediated by vitamin D receptor. Biochem Biophys Res Commun.

[52] Palmer HG, Gonzalez-Sancho JM, Espada J, Berciano MT, Puig I, Baulida J, Quintanilla M, Cano A, de Herreros AG, Lafarga M, Munoz A (2001). Vitamin D(3) promotes the differentiation of colon carcinoma cells by the induction of E-cadherin and the inhibition of beta-catenin signaling. J Cell Biol.

[53] Wietrzyk J, Opolski A, Madej J, Radzikowski C (2000). Antitumour and antimetastatic effect of genistein alone or combined with cyclophosphamide in mice transplanted with various tumours depends on the route of tumour transplantation. In Vivo.

[54] Hidalgo AA, Paredes R, Garcia VM, Flynn G, Johnson CS, Trump DL, Onate SA (2007). Altered VDR-mediated transcriptional activity in prostate cancer stroma. J Steroid Biochem Mol Biol.

[55] Spina CS, Tangpricha V, Uskokovic M, Adorinic L, Maehr H, Holick MF (2006). Vitamin D and cancer. Anticancer Res.

[56] Tocchini-Valentini G, Rochel N, Wurtz JM, Moras D (2004). Crystal structures of the vitamin D nuclear receptor liganded with the vitamin D side chain analogues calcipotriol and secocalcitol, receptor agonists of clinical importance. Insights into a structural basis for the switching of calcipotriol to a receptor antagonist by further side chain modification. J Med Chem.

[57] Matsunaga T, Yamamoto M, Mimura H, Ohta T, Kiyoki M, Ohba T, Naruchi T, Hosoi J, Kuroki T (1990). 1,24(R)-dihydroxyvitamin D3, a novel active form of vitamin D3 with high activity for inducing epidermal differentiation but decreased hypercalcemic activity. J Dermatol.

[58] Bouillon R, Allewaert K, Xiang DZ, Tan BK, van Baelen HJ (1991). Vitamin D analogs with low affinity for the vitamin D binding protein: enhanced in vitro and decreased in vivo activity. J Bone Miner Res.

[59] Xu RX, Lambert MH, Wisely BB (2004). A structural basis for constitutive activity in the human CAR/RXRalpha heterodimer. Mol Cell.

[60] Wu B, Li S, Dong D (2013). 3D structures and ligand specificities of nuclear xenobiotic receptors CAR, PXR and VDR. Drug Discov Today.

[61] Sutton AL, MacDonald PN (2003). Vitamin D: more than a ‘bone-a-fide’ hormone. Mol Endocrinol.

[62] Moreau A, Maurel P, Vilarem MJ, Pascussi JM (2007). Constitutive androstane receptor-vitamin D receptor crosstalk: consequence on CYP24 gene expression. Biochem Biophys Res Commun.

[63] Cheng HT, Chen JY, Huang YC, Chang HC, Hung WC (2006). Functional role of VDR in the activation of p27Kip1 by the VDR/Sp1 complex. J Cell Biochem.

[64] Huang YC, Chen JY, Hung WC (2004). Vitamin D3 receptor/Sp1 complex is required for the induction of p27Kip1 expression by vitamin D3. Oncogene.

[65] Diaz GD, Paraskeva C, Thomas MG, Binderup L, Hague A (2000). Apoptosis is induced by the active metabolite of vitamin D3 and its analogue EB1089 in colorectal adenoma and carcinoma cells: possible implications for prevention and therapy. Cancer Res.

[66] Koren R, Wacksberg S, Weitsman GE, Ravid A (2006). Calcitriol sensitizes colon cancer cells to H_2_O_2_-induced cytotoxicity while inhibiting caspase activation. J Steroid Biochem Mol Biol.

[67] Meng J, Zhang HH, Zhou CX, Li C, Zhang F, Mei QB (2012). The histone deacetylase inhibitor trichostatin A induces cell cycle arrest and apoptosis in colorectal cancer cells via p53-dependent and -independent pathways. Oncol Rep.

[68] Stambolsky P, Tabach Y, Fontemaggi G, Weisz L, Maor-Aloni R, Siegfried Z, Shiff I, Kogan I, Shay M, Kalo E, Blandino G, Simon I, Oren M, Rotter V (2010). Modulation of the vitamin D3 response by cancer-associated mutant p53. Cancer Cell.

[69] Longley DB, Harkin DP, Johnston PG (2003). 5-fluorouracil: mechanisms of action and clinical strategies. Nat Rev Cancer.

[70] Shah MA, Schwartz GK (2001). Cell cycle-mediated drug resistance: an emerging concept in cancer therapy. Clin Cancer Res.

[71] Mader RM, Muler M, Steger G (1998). Resistance to 5-fluorouracil. Gen Pharmacol.

[72] Takagi K, Sowa Y, Cevik OM, Nakanishi R, Sakai T (2008). CDK inhibitor enhances the sensitivity to 5-fluorouracil in colorectal cancer cells. Int J Oncol.

[73] Watanabe M, Sowa Y, Yogosawa M, Sakai T (2013). Novel MEK inhibitor trametinib and other retinoblastoma gene (RB)-reactivating agents enhance efficacy of 5-fluorouracil on human colon cancer cells. Cancer Sci.

